# Public-Private or Master-Servant? Examining the Implementation of the Serious Disease Insurance Scheme in China

**DOI:** 10.3390/ijerph17051490

**Published:** 2020-02-26

**Authors:** Yingying Ma, Zhuojun Liu, Shuguang Shen

**Affiliations:** 1 College of Public Management, South China Agricultural University, Guangzhou 510642, China; mayying@scau.edu.cn; 2 Lingnan (University) College, Sun Yat-Sen University, Guangzhou 510275, China; ssg84114060@163.com

**Keywords:** serious disease insurance scheme, public–private partnership, basic medical insurance, China

## Abstract

China’s Serious Disease Insurance Scheme (SDIS) was set up to relieve the financial burdens on serious disease patients. It is a crucial part of the national basic medical insurance scheme, which is regarded as one of the largest government-funded social security programs in the world. The most significant institutional innovation of the SDIS is that the approach of a public–private partnership (PPP) is applied in an attempt to facilitate the efficiency of its implementation. The objective of this paper is to evaluate the implementation of the SDIS in China through PPPs, and to identify the problems to be tackled if the Chinese government intends to make such a plan work better for the majority of urban and rural residents. With the effective support from local officials and practitioners, the authors of this paper collected copies of SDIS contracts of multiple cities in Guangdong, one of the most developed provinces of China. Guided by a research framework drawn from the PPP literature, details of contract enforcement were also examined. The authors discovered that the role of local states is rather dominant; they have manipulated contract drafting and implementation. Additionally, current mechanisms for profit sharing, risk sharing, and information exchange have placed insurance companies in a rather disadvantageous situation. To achieve the sustainable development of the SDIS, the authors suggest that a further reform on implementation of a PPP must be pushed forward.

## 1. Introduction

In the West, reforms on health systems are continuously conducted to enhance efficiency and improve resource allocation. In 1991, market mechanisms were introduced to the National Health Service (NHS) of the UK in pursuit of a better use of public funding. However, the effects were rather limited, as some essential conditions that are required for a market to operate were missing [1,2]. In the US, the implementation of the Patient Protection and Affordable Care Act (PPACA) significantly expanded the coverage of the country’s public healthcare system in an attempt to reduce the number of uninsured at the cost of increasing the deficit. It has also been shown that health expenditures have substantially increased since the statute was enacted [3,4,5]. Lessons from the UK and the US imply that it is quite difficult for the government to provide “cheaper” and “better” healthcare services.

In recent years, the development of the Serious Disease Insurance Scheme (SDIS) via the approach of a public–private partnership (PPP) has become a primary part of China’s social and economic policy. It was praised by the domestic media that one of the substantial achievements of China’s development was the establishment of a government-funded medical insurance scheme. According to the Ministry of Human Resources and Social Security (MHRSS), universal coverage of basic medical insurance has been provided to all rural and urban residents since 2016 (MHRSS, 2017). The healthcare safety net consists of the Urban Employee Essential Medical Scheme (UEEMS) and the Urban and Rural Resident Basic Medical Insurance Scheme (URRBMIS). The former is provided to urban workers who are hired by various types of organizations such as enterprises, governmental sectors, and non-governmental organizations. The latter combined the previous “Urban Resident Basic Medical Insurance” and “New Rural Cooperative Medical Scheme”, which, respectively, served the nonworking urban residents and peasants. Previously, only basic medical services were covered by UEEMS and URRBMIS. In 2016, Keqiang Li, Premier of the State Council, declared that a “serious disease insurance scheme” would also be included into the basic medical insurance when he was delivering a government work report at the annual parliamentary session. As a result of the upgrade, China’s basic medical insurance has become the largest public insurance program in the world, as it covers more than 1.3 billion of the country’s population and has substantially increased the expenditure by incorporating the SDIS. As an extension of the basic medical insurance, the SDIS was established to prevent more people from “falling back to poverty due to serious diseases (yin bing zhi pin)”. In practice, the SDIS purchases insurance services from commercial insurance companies with a portion of basic medical insurance surplus. Once a qualified insuree is diagnosed with a listed serious disease, he or she can apply for reimbursement for a certain proportion of the medical expenses. According to the Premier, by the end of 2017, 17 million people had settled claims successfully under the SDIS, and such a number was set to rise to 20 million by the end of 2018 [6]. By handling such a large amount of funding, are the governmental sectors and their partners getting along well with each other? It is rather necessary to examine whether the PPP approaches are well utilized during the process of enforcing the policy of the SDIS and relieving financial burdens on Chinese patients?

In China’s social security system, two major forms of PPP are widely applied. The first form is that the government sells franchises to private insurance companies, allowing them to sell basic compulsory insurance to the citizens. The second form is to outsource the whole package of a certain kind of insurance, while the government plays such roles as funder, rule-maker, or supervisor [7]. However, existing research on PPP projects has overlooked several crucial issues. First of all, there are not enough detailed case studies highlighting the exact roles played by the public and non-public sectors and evaluating the quality of cooperation during the implementation of the SDIS. Second, not much attention has been paid to the financing element of healthcare services in developing countries. There are plenty of studies focusing on PPP projects in the West, while research on developing countries is scarce.

The purpose of this paper is to evaluate the implementation of the SDIS in China through a PPP, and to identify the problems to be tackled if the Chinese government intends to make such a plan work better for the majority of urban and rural residents. It is still unclear whether the government and the insurance companies are working closely enough and whether the SDIS has made achievements as expected by the country’s leadership. Among all provinces, Guangdong is the earliest one to have completed setting up its own SDIS. According to our case studies, more than 70 million people had joined the scheme by the end of 2014, and SDISs are conducted via a PPP in 18 prefectures, accounting for more than 85% of the cities in the province. Based on the fieldwork supported by the provincial and local officials, the authors of this paper successfully collected all of the current SDIS contracts in Guangdong, which were signed between insurance companies and local governments. Findings of this paper are based on the reviews of the contract details and their enforcement.

The rest of this paper is divided into five parts. In the following section, we briefly review the literature on the PPP theory and generate an applicable analytical framework for this paper. The third section provides an overview of SDIS implementation in China and Guangdong Province; the research methods are also introduced. The fourth section provides empirical evidence to evaluate the implementation of the SDIS. The fifth part discusses the results presented, and the last part concludes the paper.

## 2. Literature Review and Analytical Framework

### 2.1. Public-Private Partnerships: Concept and Application

The accurate definition of a PPP remains controversial. Scholars summarize that participants of a PPP include governmental departments, citizens and volunteers, private firms, and non-profit organizations (NPOs) [8,9]. However, there is no concrete answer explaining what kind of partnerships are counted as genuine in the PPP model. Some scholars deem that the term PPP normally refers to dynamic interactions between government and private sectors [10]. Through his widely read book, Savas argues that a PPP refers to any kind of production and provision of public goods organized by both public and private sectors [11]. However, the definition from the International Monetary Fund (IMF) is more aggressive, which puts forward that a PPP is a contract which allows private sectors to take the role of public sectors and to provide a certain kind of public service [12]. Since the late 1990s, traditional forms such as privatization and direct investment from the government have been replaced by PPPs in the field of public goods provision and infrastructure building [13,14]. Differentiated from full privatization, it is advocated by some other scholars that a PPP is suitable to preserve the government’s ownership over public goods, while efficiency of operation can also be enhanced [15]. Moreover, a PPP is also regarded as an alternative way under some circumstances, while the process of privatization is hindered [16]. In this paper, a relatively broad definition of PPPs is applied, which refers to the general cooperation on fund raising and management of public services [11,17].

The necessity and incentive of PPPs is another hot topic to be discussed. It is advocated by scholars that private firms and NPOs, which normally have stronger economic and social motivations, could be much more efficient than governmental departments [9]. The mechanism of sharing also plays an important role. Local governments, private firms, and NPOs are able to share technologies and other resources via a PPP. Meanwhile, the building of good partnerships would be a reliable method to spread the risks [18]. The implementation of a PPP can even improve local governance substantially [19]. On the one hand, inviting more governing bodies to participate in dealing with local affairs can further push forward the administrative reform, making them pay more attention to policy making rather than detailed management. On the other hand, social organization would have more chances to develop. It is a general consensus that a “win-win” situation is likely to be achieved if a PPP is conducted properly.

PPPs were not frequently discussed in the field of healthcare until the late 1990s. Scholars tend to pay attention to the effectiveness, benefits, public interests, efficiency, and partnership of PPPs [20]. Among all of these factors, the relationship between the public and private sectors remains central. Instead of weakening the role of the government, it is advocated that public sectors should improve health services in innovative ways and continue to act as regulators [21]. In the discussion on the UK’s Public Health Responsibility Deal, scholars argue that the impact of PPPs is limited because the government only uses voluntary agreements as a key strategy for encouraging corporate action in public health. They suggest that formal incentives and sanctions should also be built accordingly [22]. Some other studies focus on PPPs’ application on healthcare infrastructure building, while projects in multiple countries have been investigated. Evidence from Portugal’s hospitals indicates that PPP hospitals and public hospitals provide healthcare services with similar quality and access [23]. Another study based on a single case in the New Mestre Hospital of Italy suggests that PPP projects should also take into account the risk of uncertainty, the beneficial effect on health services, and the interests of the general public [24]. As a flexible approach to mobilize non-public sectors and resources, PPPs have already achieved a certain degree of success in providing healthcare services.

In terms of the application of PPPs in China, scholars mainly focus on the relationship between state and social organizations. Ideally, researchers deem that the government should be a smart buyer and a sophisticated supervisor in the purchase of public goods [25]. However, as the Chinese government has long been the dominant party in governance, cooperation between the state and society is usually weak and vulnerable [26]. There is an insufficient level of trust in the partnerships, and as a result, public sectors tend to manipulate social organizations instead of treating them as close partners [27]. Furthermore, the scale of social organizations in contemporary China is generally small. The only way for them to survive is to heavily rely on government-funded projects [28]. Therefore, Chinese scholars argue that there is still a long way to go to realize “fair play” in China’s PPP projects [29]. To acquire equal status in cooperation, social organizations need to make much greater efforts [30]. Thus, the state remains the dominator in China’s PPP projects [31].

### 2.2. Gaps in Research and Analytical Framework

Current literature has pointed out that PPPs are a promising way to enhance the efficiency and quality in providing healthcare services. However, several gaps are waiting to be filled. First of all, previous research has overlooked the importance of good relationships between the government and non-public sectors in the field of healthcare. Scholars confirm that the government is too strong to maintain good partnerships with social organizations in China, while only a few of them pay attention to the healthcare PPP projects. Second, only a limited number of studies concentrate on the application of PPPs in healthcare insurance. Financing is one of the crucial parts in healthcare services, yet there is an insufficient number of studies that can elaborate on how the approach of PPPs performs in building the insurance system. Third, existing studies have provided plenty of cases in developed countries, while empirical details of developing countries are rare. There is a pressing need to identify the effectiveness of PPPs in healthcare services in China, the largest developing country of the world.

To fill the aforementioned gaps, this paper discusses the implementation of the SDIS in Guangdong by reviewing the specific contracts signed by the prefecture-level governments, alongside their subsequent enforcement. Since our analysis mainly depends on the copies of contracts and interviews with local officials, insurance company staff, and scholars, the relational aspect of partnership is emphasized. According to previous studies, the characteristics of PPPs include cooperation; durable relationships; development of mutual products/services; sharing of risks, costs, and benefits; and mutual value addition—most of which are closely related to the relational aspect [32]. The results of a survey based on Dutch PPP projects suggest that both trust and management correlate significantly with perceived performance, although more case studies are needed to further consolidate these findings [33]. It is also discussed by some other papers that the quality of the contract, interaction, managerial activities, and trust jointly steer the performance of PPPs [34,35]. Having derived key components of the relational aspect from existing literature, an analytical framework is applied. It tends to provide updated analysis to verify whether a good relationship between the public and non-public sectors is the key component. Combing the factors from previous research and the features of our data, we formed an analytical framework with equal status, profit sharing, risk sharing, and information exchange. First of all, we wanted to figure out whether equal status between the government and non-governmental sectors is the key to successful PPP implementation, as stated in previous research [11,13,36]. Signing a long-term and fair contract is the embodiment of such an attribute [37]. Second, this paper also aimed to clarify the significance of profit sharing and risk sharing. It is recommended by scholars that the government should allow non-governmental sectors to have a reasonable cut of the revenue and share part of the risks [38,39,40]. Last but not least, we also examined the impact of information exchange and determined whether high transparency of information is needed to improve the cooperation [41,42]. Table 1 shows the attributes of the above framework and the proposed characteristics of successful PPP project implementation. Section 4 is organized in accordance with this framework.

## 3. Materials and Methods

### 3.1. Study Area

This study is mainly based on the implementation of the SDIS in Guangdong Province. Under the documents and guidelines prepared at the national level, provincial and prefecture-level governments began to explore the idea of adopting PPPs while setting up the SDIS. By mid-2014, 260 prefecture-level cities of 26 provinces had built up their own SDISs, covering up to 470 million people in the country [43].

Among all of the provinces, Guangdong was the first province to launch the SDIS in China. In 2009, Zhanjiang became the first city that provided serious disease insurance to the citizens jointly by the local government and commercial insurance companies. In a short period of time, attention from researchers and the leadership was drawn to the innovative experiment of Zhanjiang. By the end of 2012, four other cities in Guangdong, including Shantou, Zhaoqing, Qingyuan, and Yunfu were designated as pilot cities, applying the PPP approach to the operation of basic medical insurance schemes. One year later, SDISs were established in these places, while another 16 prefectures also promulgated planning documents for SDISs. According to the data collected from our fieldwork, 18 prefecture-level cities out of 21 in Guangdong decided to use the PPP approach to set up SDISs. In 2014 alone, more than 70 million of those insured had joined the scheme and 407,000 people had settled their claims. In spite of the rapid development, our data show that there were deficits in 12 cities’ SDISs in 2014, accounting for two-thirds of the total number of prefectures. Does this imply that PPPs are not a suitable approach for SDISs? Using detailed evidence, Section 4 establishes the answer to this question.

### 3.2. Data Sources and Methodology

In this paper, the implementation of the SDIS was examined using the analytical framework comprised of contract status, profit-sharing mechanism, risk-sharing mechanism, and information exchange mechanism. One of the greatest difficulties of research on PPPs is data collection at the local level. Previously, contracts signed between the government and contractors were usually confidential. It was almost impossible to collect a true copy to support academic arguments. Having benefited from the policy of information disclosure, official documents like bid invitations and clauses of the PPP contracts are gradually becoming open to the general public. Although some information may only be available online for a very short period, it still greatly enhances the accessibility of SDIS data. For instance, the city of Zhaoqing located in western Guangdong issued an SDIS bid invitation in early 2019 via the online platform built by the Ministry of Finance. The invitation specified details such as budget, needs, and bidder qualifications. Similar websites of other cities or provinces can also be found easily online. Not only in prefecture-level cities, but also some county-level cities invite SDIS bidders through the same platform. Another advantage of data collection, particularly in this study, is the authors’ close relationships with officials and representatives from both the governments and enterprises, which makes in-depth interviews with them rather convenient to be conducted. In total, the authors interviewed 24 stakeholders, including 16 officials from Guangdong provincial government and nine prefecture-level cities, five senior staff from four major insurance companies, and three researchers in this field. These three researchers are, respectively, from the Institute of Social Security affiliated to the Ministry of Human Resources and Social Security, and the top universities in Guangdong Province and Zhejiang Province. They made suggestions as to how this study could be conducted and provided valuable insights into SDIS from both national and local perspectives. Several informants of this research were also recommended by them.

Specifically, this research was carried out as follows. First, we searched for and downloaded bid invitations and contract templates from Guangdong Public Resources Trading Center and local Human Resources and Social Security Bureaus (HRSSBs). Contracts and documents from 18 cities were collected. A preliminary judgement of contract status was formed at this stage. After the thorough review of documents was finished, we invited stakeholders of SDISs to have in-depth interviews and verified our findings. Semi-structural interviews and participant observation were the main techniques used. The authors were also invited to participate in a number of meetings and to provide suggestions as third-party experts. The major meetings are including two seminars on SDIS organized by the Human Resources and Social Security Department of Guangdong Province, negotiation meetings between local governments and insurance companies in two prefecture-level cities and two symposiums organized by Guangdong Social Insurance Association. As the authors are not affiliated to, or funded by the above governmental departments or organizations, we are capable of giving advice on how to promote and improve the implementation of SDIS from a neutral point of view. The documents and notes collected were reorganized and sorted out manually or by software (i.e., NVivo 10 manufactured by QSR International based in Melbourne, Australia). The selected key findings and arguments are presented in the following sections. To protect the interviewees’ identities, all of their personal information was removed. The names of the related cities were replaced by codes (i.e., A City, B City) to make sure that the sensitive information is untraceable.

## 4. Results

### 4.1. Assessing the Statuses in Contracts

In a healthy relationship, the statuses of HRSSBs and insurance companies should be equal, based on the fair clauses in the contracts. Yet the real story was not told as expected. Our fieldwork indicates that the HRSSBs tend to have full control in the processes of contract preparation and execution.

In terms of contract drafting, the cities’ HRSSBs are the dominant actors. All of the rules and details are decided by the local officials. In practice, the government does not care much about the professional qualifications of insurance companies. Instead, they only pay attention to how low their quotations can be. As long as the price is low enough, a bidder will possibly win the competition. In one of our interviews, an associate department head of a HRSSB said:

*“Current bid invitations of SDIS and contracts are all prepared by the local government sectors. Details including management cost, profit rate and even staff headcount are prescribed rigidly. In some cities, communication between government and insurance companies may be better than others, but it does not make any substantial differences.”*

(Interview Number: BJ02092401)

The length of the contract is another critical factor. According to other scholars, contract length reflects the degree of trust between the partners [13,44]. An SDIS is a rather long-term project. On the one hand, serious diseases do not only affect a few years of the insured’s life, but rather, they could be incidents scattered in multiple life stages of the patients. On the other hand, insurance companies will inevitably take a considerably long period of time to recover their costs. Hence, only contracts covering a sufficient number of years can ensure the smooth and stable implementation of SDISs. According to the national and provincial documents of Guangdong, the minimum length of an SDIS contract is three years. Nonetheless, not all of the cities intend to comply with the regulations. Referring to the data shown in Table 2, the HRSSB of Shaoguan refuses to provide a contract with a standardized length. Rather, only a two-year-long option is available to the bid winner. The majority (12) of the cities offer a three-year contract, which merely meets the minimum requirement. Only four cities (Shantou, Meizhou, Shanwei, and Zhanjiang) intend to sign a four- or five-year contract with the companies.

Signing short-term contracts is rather harmful to PPPs. For insurance companies, deficit becomes almost unavoidable. To cut the loss, they can only invest limited resources and manpower into SDISs, jeopardizing the interests of the common insured. For the governmental departments, frequent changes of partner also significantly increase managerial cost and uncertainty.

In addition to the contract drafting and contract length, execution of contracts also restricts the operation of the insurance companies. Savas advocates that enough flexibility should be left to the contractors in order to encourage them to innovate [11]. Instead of following this principle, the HRSSBs of 11 cities have made rigorous clauses in SDIS contracts, stipulating that staff assigned to the SDIS must meet a number of criteria such as scale, staff’s professional ranking, educational background, and salary. In some contracts, even the use of cars is regulated. The SDIS contract of the A City states that the insurance company should allocate 219 staff to the project, 144 of whom should have an educational background related to medical care or pharmacy. In the contract prepared by F City, the numbers of desktop computers, printers, photocopiers, and fax machines are all set by the local HRSSB. Although rigid management is able to prevent some risks, it also snuffs out the companies’ incentive to innovate and improve their services.

### 4.2. Assessing the Profit-Sharing Mechanism

A rational profit-sharing mechanism is the key component to the building of sustainable partnerships between the public and private sectors [40]. Documents issued by the central and Guangdong provincial governments specify that the suggested profit rate of SDISs is “to break even (baoben)” or “to pursue meager profit (weili)”. In practice, these guidelines are rather ambiguous and the local HRSSBs interpret the documents quite differently from the insurance companies. Local HRSSBs deem that the managerial cost of the insurance companies should be largely borne by the insurers. Only when there is a profit generated from the SDIS can the insurance companies draw a small portion as compensation for the costs. On the other side, insurance companies argue that their costs consist of two parts: settlement cost and management cost. The former is determined by the actual number of claims from the insured and the latter refers to all of the expenses of daily operations, including manpower, hardware, software, case investigation, promotion, staff training, and so forth. As the amount of management cost is relatively fixed, insurance companies assert that the local government should be responsible to this part, even when the SDIS makes a loss. In one of our interviews, a department head from A City admitted:

*“The existing deal in our city is rather harsh. The management fee of the insurance company is six million yuan per year. I am sure that they cannot break even. According to the contract, they should assign 200 staff to handle SDIS. The maximum profit rate they can reach is 4%, which is definitely less than six million. As a result, they come to me every day and tell me how difficult they feel.”*

(Interview Number: RS02091101)

The breakdown of management cost is also controversial. The China Insurance Regulatory Commission merely stipulates that manpower cost and promotion cost can be covered by the funding for SDISs; temporary costs, including software maintenance cost, staff training cost, and consultation cost, cannot be taken into account. In some cities, these kinds of costs can account for up to 90% of the total management cost. A department head of a large state-owned insurance company stated to us:

*“We have hired a large number of staff specifically for SDIS. The burden of our human resource department has been increased substantially. The building of information system for SDIS also costs a fortune. To be fair, I propose that all these expenses should be taken into account as formal management cost approved by the government.”*

(Interview Number: SYBX01102001)

Even though an SDIS is not a profit-seeking project, it is rather necessary to review the rationale of the current calculation method of cost-bearing and profit-sharing. In the cases of Guangdong, deficits are commonly seen in most of the SDISs. To motivate the insurance companies, a more competitive rate of profit should be put forward.

### 4.3. Assessing the Risk-Sharing Mechanism

The major risk of implementing SDISs is the potential deficit. At the provincial level, only a broad guideline is provided by the Implementation Scheme of Serious Disease Insurance of Guangdong (Guangdong dabing baoxian shishi fangan). It stipulates that deficits brought by state policies (zhengcexing kuisun) should be handled according to the contract, while other kinds of deficits (feizhengcexing kuisun) should be undertaken by the insurance companies. At present, there are four types of risk-sharing or deficit-sharing mechanisms in Guangdong. As Table 3 presents, cities such as Guangzhou have set up a fixed deficit rate (i.e., 4%), within which the government and the insurance companies respectively bear half of the losses. If the deficit rate is higher than the fixed rate, the insurance companies are responsible for covering the extra losses. Heyuan, Yangjiang, and Qingyuan allow the insurance companies to adjust the premium in the following year when the deficit is recorded in the current year. However, they should also fully undertake the existing losses. In Huizhou and Shanwei, insurers are permitted to adjust the premium only when the deficit rate is higher than a certain level (i.e., 5%–10%). The HRSSBs of Shantou, Foshan, Shaoguan, Chaozhou, and Yunfu vaguely state that they comply with the provincial guideline. The rest of the cities (Meizhou, Zhaoqing, Jieyang) have not yet made relevant arrangements for risk sharing.

The existing flawed deficit-sharing mechanism fails to provide sufficient protection to the insurance companies. Referring to our fieldwork, deficits of SDISs were recorded in 10 cities of Guangdong in 2014. The total loss was 133.95 million yuan. Only 20.53 million yuan (15.3%) of the total loss was covered by the local governments. Seven cities did not pay any money for the loss. The rest of the deficits (CNY 113.42 million, accounting for 84.7%) were undertaken by the insurance companies. Technically, even loss covered by the government is undertaken by the basic medical insurance, which is actually supported by the insured themselves. In this manner, the local government takes no responsibility at all, whereas the allocation of deficits of SDISs indicates that insurance companies take too much.

### 4.4. Assessing the Information Exchange Mechanism

Information exchange is crucial to the success of PPPs [41,42]. The critical technique of an insurance company is the skill of actuarial analysis. As we all know, an SDIS can achieve its goals more easily with the insurers’ professional services. However, keen competition in the market results in asymmetric and incomplete information [45]. The self-protection mechanism of competitors would seriously impede the process of information sharing and exchange. In terms of SDIS implementation, there are three reasons to explain why insurance companies find it so difficult to obtain the necessary information.

First, the local HRSSBs refuse to provide the historical data of medical insurance programs. Actuarial analysis relies heavily on accurate information of the past. It is a technique based on the assessment of “big data”. Without the help from local officials, insurance companies can merely estimate and figure out a rather rough number of premiums, which could significantly deviate from reality.

Second, information sharing is against the current legal arrangements. According to the current version of Social Security Law, public sectors are prohibited from leaking personal information of the insured (Article 81). As a result, it is impossible to set up an information exchange mechanism between local HRSSBs and insurance companies. The associate head of C City’s HRSSB stated:

*“Social insurance information system contains a vast amount of personal information of all the insured. Protecting it is one of the most important jobs of both national and local governments. Unless the higher level orders us to do so, we will never sharing any of personal information with insurance companies.”*

(Interview Number: RS02091102)

Third, insurance companies have no right to inspect the operational details of SIDSs. In most of the projects, SDISs are associated with the basic medical insurance. Therefore, inspection of the operation of SDISs can only conducted by HRSSD officials. Staff from insurance companies are forbidden to join. A department head of A City explained:

*“The insurance company wanted to inspect our data of basic medical insurance. We turned them down for two reasons. First, we believe that they should contact the hospital instead of us to acquire data. Second, according to the signed contracts, it is already a mutual agreement that SDIS should settle the claims once a case is qualified. We are the ‘accountants’ and they are the ‘cashiers’. They should follow our orders and trust our decision. We will not share out basic medical insurance data with them.”*

(Interview Number: RS02091103)

## 5. Discussion

### 5.1. Overall Evaluation of SDIS Implementation

According to the evidence above, SDISs are an extension of basic medical insurance and are set up with the PPP approach to financially protect Chinese insurees from serious diseases. The growing number of settled claims in recent years indicates that SDISs are rather successful on the demand side. However, our study argues that the performance of the supply side is far from satisfactory due to the unfavorable relationship between the local state and insurance companies. We established and applied an analytical framework to evaluate SDIS implementation in Guangdong and discovered that the status of the local state and insurance companies is not equal under the PPP. The latter can only take orders from the state and they must follow every detailed rule. They also bear most of the risks while they can only share a small part of the profit. In terms of information exchange, insurance companies cannot gain enough data for basic operation. In the relational aspect of PPPs, we argue that the major constraint of SDIS implementation at the current stage is the predominance of the local government and the lack of trust. It appears that a de facto “master–servant” type of relationship between the local government and the insurance companies has formed. Having revisited some of the key officials and insurance company staff, we identified two major explanations, which will be elaborated in the following subsections.

### 5.2. Local HRSSBs’ Strong Motivation to Control

The local governments have extremely strong motivation to prevent insurance companies from becoming involved in the core business of SDISs. Scholars argues that social security system reform is a process to find an optimal combination of the state and the market [7]. In 2009, the National Medical System Reform Scheme promulgated by the State Council already encouraged governmental sectors to cooperate with non-governmental sectors via outsourcing and building up PPPs. From 2012 to 2015, a series of documents were issued by the State Council and relevant ministries to facilitate the trials of applying PPPs as a means to set up SDISs. Nowadays, basic medical insurance is fully run by the local HRSSBs. From the perspective of Central government, they are planning to transfer the whole basic medical insurance system to non-governmental sectors in the near future. Some provinces such as Anhui have already launched the reform. For many other provinces, using PPPs in SDISs may be the first step of further reform and an experiment on cooperating with non-governmental sectors. Therefore, the establishment of SDISs concerns not only the well-being of the citizens, but also the success of the national medical system reform.

In this case, what is the opinion of local HRSSBs? In cities where HRSSBs are understaffed, insurance companies are quite welcome by the officials. The SDIS of Z City is a vivid example. One of their officers said to us:

*“In 2009, due to the combination of UEEMS and URRBMIS, the population managed by us had increased from 580,000 to 5.46 million. At that time, we were underfinanced and understaffed. People’s Insurance Company of China (PICC) contacted us and proposed that they could set up SDIS. As the result of negotiations, we agreed that 15% of basic medical insurance premium of Z City would be used to purchase serious disease insurance from PICC. With their help, we can handle a larger amount of work without increasing the headcount of our staff.”*

(Interview Number: RS02091604)

However, HRSSBs and insurance companies do not get along well in many other cities. According to the findings from previous studies, bureaucracies inherently tend to maximize their budget [46]. They have a strong motivation for obtaining more power, a larger budget, and more staff [47]. With a larger size, a governmental department can expand its authority and draw more resources from a higher level. According to the number provided by the National Healthcare Security Administration, the surplus of basic medical insurance is 1.61 trillion yuan, all of which is potential funding that could be mobilized by SDISs [48]. With their possession of the control over the funding, local HRSSBs are allowed to employ more staff and upgrade the facilities of the offices. Hence, the control over SDISs is closely related to the accessibility to power and interest. Moreover, dominating the operation of SDISs can also help local HRSSBs to better manage the hospitals within their jurisdiction. Presently, government-funded medical insurances are the hospitals’ most important income sources. Using SDISs as a bargaining tool, HRSSBs are able to exert greater influence on setting the prices of medical services.

From the perspective of many HRSSB officials, whether the approach of PPPs should be introduced remains rather controversial. Opponents deem that insurance companies merely want to take a share of the profit generated by SDISs. Referring to a high-level official, local HRSSBs are absolutely capable of handling SDISs by themselves without using additional facilities or hiring more staff [49]. In other words, some officials believe that it is rather unnecessary to purchase services from the companies. Besides, governmental sectors are much more familiar with the policies and they can finish the tasks at a lower cost [50]. Even though documents from the central state have stipulated that PPPs are a preferred and recommended approach, some local governments still insist on operating SDISs with their own HRSSB. As an associate head of HRSSB of A City said:

*“Whether to invite insurance companies to SDIS is a case-by-case topic. If the local HRSSB is too small to handle it, using PPP is understandable. But the case is different in our city. When we invited PICC in 2014, we actually had a large team of staff who can deal with all kinds of SDIS matter. We agreed to use PPP only because it was the policy actively promoted by the central and provincial governments.”*

(Interview Number: RS02072805)

### 5.3. Insurance Companies’ Enthusiasm to Take Part

Commercial insurance companies are enthusiastic to take part in SDISs because they have set their sights on long-term goals. They decide to pay more attention to acquiring larger future development space rather than short-term interests. With this principle, insurance companies do not refuse to operate defective SDIS projects. From their point of view, being involved in government-funded projects offers them the chance to get in touch with more potential clients and explore more business opportunities, just as a project manager said:

*“We believe that SDIS is definitely not the only insurance program that conducted by PPP approach. We can learn from Premier Li’s report that market mechanisms will be introduced to much more social security projects. For companies like us, operating SDIS is a process of building trust with government officials and fellow citizens. When the insured want to purchase other insurance programs or the local governments plan to launch another social insurance project, we are likely to be selected and become their partners again.”*

(Interview Number: SYBX01102003)

Another reason for the companies’ enthusiasm is the accessibility to valuable data. Compared with developed countries, the Chinese insurance industry lacks reliable data and experience. This situation has severely limited the development of nearly all insurance companies. SDISs provide an option to change the status quo. By sorting out the information of the insured, insurers can design and develop more suitable plans for their Chinese clients. For the larger companies such as PICC and Pingan, operating SDISs can also optimize their decisions on selecting the location of branches, and on allocation of staff. An officer from E City had seen it through and said to us:

*“The main objective of our partners from insurance companies is to access to our data. In the near future, they may seek for more chances to set foot in the detailed management of basic medical insurance. To achieve this goal, they are willing to undertake current losses. In the long run, they will absolutely benefit from it.”*

(Interview Number: RS02072806)

In summary, although the central government actively promotes SDISs conducted through the PPP approach, many of the local states merely react passively. The principles of PPPs have been severely twisted in multiple cases. Local HRSSBs only regard insurance companies as a tool to relieve their financial burden, while they have made great efforts to keep the companies away from the center of their turf-power, funding, and information. According to our examination on the implementation of SDISs in Guangdong, the de facto “master-servant” relationship between local HRSSBs and local insurance companies has caused four aspects of damage. For the local HRSSBs, the morale of officials is generally low, as they should maintain the control over SDISs and always take precautions against the insurance companies. For the insurers, the effectiveness of participating in SDISs is always questionable. The pursuit of profit is at variance with deficits generated by SDISs. At some point, it could be rather difficult for the insurers to decide whether they should continue to invest in SDISs, regardless of the growing cost. In addition to impairing the “master” and the “servant”, collateral damages are also suffered by hospitals and patients. In the past, only the local Health Commissions (former Health Bureau) and HRSSBs were responsible for regulating the operation of hospitals. Currently, the insurance companies are encouraged by the central government to engage in controlling medical expenses. Even though insurance companies have no genuine authority at this stage, taking orders from one more regulator is the last thing that hospitals are willing to do in the future. As the insured of SDISs, patients are supposed to be the major beneficiaries, especially the poor. Nevertheless, since the insurance companies have not yet acquired sufficient clearances to access to the data they need, the process of settling claims via an SDIS could be long and tedious. If the aforementioned unfavorable circumstances do not improve as soon as possible, PPPs based on SDISs are likely to break down at some point in the future.

## 6. Conclusions

Relying on the national expansion of basic medical insurance, serious diseases insurance has nominally covered every Chinese citizen. The number of claims settled by SDISs has been growing every year since its large-scale implementation. As the pilot province of most of the reforms, Guangdong was the first place to launch an SDIS and applied PPPs as the major operational approach. The purposes of using PPPs in SDISs include enhancing efficiency, encouraging institutional innovation, and improving the state-society relationship. This idea is similar to the one advocated by the “Reinventing Government” movement in the US, which believes that the government should “steer, not row, the boat” [51,52].

The findings of this paper are against the above vision. The authors thoroughly reviewed the accessible SDIS contracts of 18 cities in Guangdong. The details of contract enforcement were also investigated through interviews with local officials, insurance company staff, and scholars. We realized that the relationship between the local government and the insurance companies is of a de facto “mater–servant” type rather than an equal “public-private” partnership. Such a summary is based on four findings. First, local governments have manipulated contract drafting and enforcement; thus, the status of insurance companies becomes rather subordinate. Second, insurers are merely allowed to pursue meager profit; it is impossible to yield a considerable amount of income from SDISs. Third, insurers undertake nearly all of the risks. Following the provincial guidelines and contracts, it is always the insurance companies that are responsible for covering the majority of the losses incurred by SDISs. Last but not least, information exchange is blocked by the local state. Therefore, insurance companies have no choice but to operate SDISs blindly without sufficient data. Substantial evidence indicates that the twisted public–private partnership has jeopardized the further development of SDISs. To improve government-funded medical insurance services, a more fair and equal status between China’s local state and insurance companies is awaiting to be built.

The findings of this paper confirm the importance of the relational aspect in PPP operation. As the literature suggests, we also found that the performance of SDISs heavily depends on the quality of the contract, interaction, managerial activities, and trust between both parties [34,35]. The authors of this paper were delighted to see that some positive changes have been made. Due to the recent administrative restructuring, SDISs are now transferred from the HRSSBs to the Healthcare Security Bureaus (yiliao baozhang ju; HSBs), which were originally established to focus on the management of China’s healthcare system. Meanwhile, as most of the SDIS contracts in Guangdong are going to expire, local HSBs are also working on the extension of existing contracts. According to our recent investigations, most of the HSBs are willing to continue the cooperation with their current partners, demonstrating that the approach of PPPs in SIDSs has made a certain degree of progress. More trust has been built between the local states and insurance companies. However, to realize the sustainable development of SDISs, further reform on profit sharing, risk sharing, and information exchange must be pushed forward. We suggest that future research take some of these aspects as focal points to conduct deeper analysis.

We also admit that this study has some limitations. Since our analytical framework is mainly based on the relational aspect of PPPs, proper adjustments are needed when it is applied to cases other than SDISs. Moreover, although we took most of the cities in Guangdong Province into account, concentrating on a handful cases may not be able to reveal the whole picture of SDISs in China. Comparisons offered by future studies would definitely improve the findings of this paper.

## Figures and Tables

**Table 1:** Attributes of successful public-private partnership (PPP) project implementation.

Attribute	Characteristics Included	Sources
Equal Status	Long-term and fair contracts	[11,13,36,37]
Profit Sharing	Stable return of investment	[39,40]
Risk Sharing	Reasonable risk-sharing mechanism	[38,40]
Information Exchange	High transparency of information	[41,42]

Source: Authors, based on references cited.

**Table 2:** Contract lengths of serious disease insurance schemes (SDISs) in different cities of Guangdong.

Length (Years)	Prefecture-Level City
2	Shaoguan
3	Guangzhou, Zhuhai, Foshan, Heyuan, Huizhou, Jiangmen, Yangjiang, Maoming, Zhaoqing, Qingyuan, Chaozhou, Jieyang
3.5	Yunfu
4	Shantou
5	Meizhou, Shanwei, Zhanjiang

Source: Human Resources and Social Security Bureaus (HRSSBs) of the above cities.

**Table 3:** Deficit-sharing mechanism of SDISs in different cities of Guangdong.

Arrangement	Prefecture-Level City
Setting up a fixed deficit rate	Guangzhou, Zhuhai, Jiangmen, Zhanjiang, Maoming
Allowing the adjustment of premium in the following year	Heyuan, Huizhou, Shanwei, Yangjiang, Qingyuan
Following the provincial guideline	Shantou, Foshan, Shaoguan, Chaozhou, Yunfu
Not stated	Meizhou, Zhaoqing, Jieyang

Source: HRSSBs of the above cities.
